# LGL-Phenocopy With Dual AV Nodal Physiology and Malignant SVT: A Diagnostic Pitfall With Sudden Deaths

**DOI:** 10.1016/j.jaccas.2026.109669

**Published:** 2026-08-10

**Authors:** Ammar Yasser Negm, Saeed Zhfan, Mohamed Khalil, Omar Hassan Ali

**Affiliations:** a Faculty of Medicine, Port Said University, Port Said, Egypt; b Faculty of Medicine, Hadramout University, Al Mukalla, Hadramout, Yemen; c Faculty of Medicine, Mansoura University, Mansoura, Egypt

**Keywords:** arrhythmogenic cardiomyopathy, dual AV nodal physiology, Hisian extrasystole, LGL-phenocopy, short PR interval, sudden cardiac death

## Abstract

**Background:**

The short PR interval without preexcitation (Lown-Ganong-Levine-phenocopy) is often considered benign, but its association with malignant substrates is underappreciated.

**Case Summary:**

A 45-year-old man with no previous cardiac history presented with palpitations, dyspnea, and diaphoresis. Electrocardiogram (ECG) during symptoms showed supraventricular tachycardia at ∼220 beats/min with a Hisian extrasystole and QRS variability. Postconversion ECG revealed short PR and dual atrioventricular nodal physiology. Echocardiography showed subtle nonterritorial hypokinesia. Family history included sudden cardiac death in 3 siblings.

**Discussion:**

This case demonstrates that Lown-Ganong-Levine-phenocopy with dual atrioventricular nodal physiology and Hisian extrasystoles can be a red flag for a lethal inherited arrhythmogenic substrate requiring comprehensive evaluation including cardiac magnetic resonance, genetic testing, and family screening.

**Take-Home Messages:**

A short PR pattern with a malignant family history warrants comprehensive evaluation. Surface ECG red flags should not be ignored even when the resting ECG appears benign.

## Visual Summary

**Figure:**
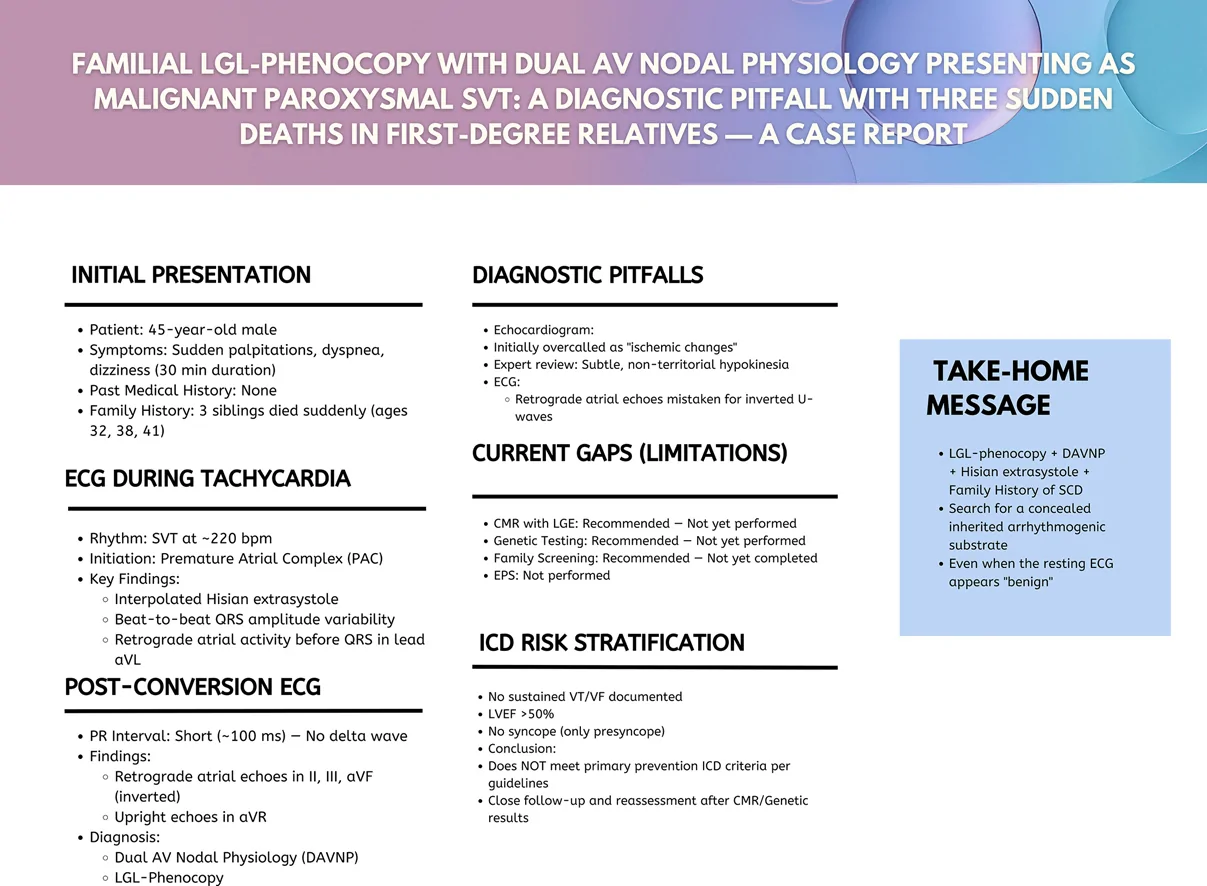

The electrocardiographic finding of a short PR interval (<120 ms) without ventricular preexcitation has been historically designated as Lown-Ganong-Levine (LGL) syndrome, a controversial entity lacking consistent anatomic or electrophysiological correlation.^1^ Modern electrophysiology has largely abandoned this syndromic label in favor of descriptive terms such as "enhanced AV nodal conduction" or "LGL-phenocopy," recognizing it as a heterogeneous conduction phenotype rather than a discrete clinical syndrome.^2^ Although often considered an incidental or benign finding, this pattern can facilitate the initiation and maintenance of supraventricular tachycardias (SVTs), particularly atrioventricular nodal re-entrant tachycardia (AVNRT).^3^

More critically, emerging evidence suggests that certain short PR phenotypes, especially when accompanied by specific electrocardiographic signatures and familial arrhythmia patterns, may serve as surface markers for concealed inherited arrhythmogenic cardiomyopathies.^4^ These conditions, which include arrhythmogenic left ventricular cardiomyopathy and desmoplakin (DSP) cardiomyopathy, carry significant risk of malignant ventricular arrhythmias and sudden cardiac death (SCD).^5^

We present a compelling case illustrating how an apparent LGL-phenocopy with dual AV nodal physiology (DAVNP) unmasked a high-risk familial arrhythmogenic substrate. This case highlights critical diagnostic pitfalls, emphasizes the invaluable role of early 12-lead electrocardiogram (ECG) documentation during symptoms, and underscores the imperative of familial evaluation when red flag features are present.

## Case Presentation

### Clinical history and presentation

A 45-year-old man presented to a primary health care center with sudden-onset palpitations of 30 minutes' duration, accompanied by dyspnea, dizziness, and diaphoresis. He reported no chest pain, syncope, or prior similar episodes. His medical history was unremarkable for cardiac disease, hypertension, or diabetes. He was a current smoker and reported recreational qat use.

Crucially, the family history was profound: 3 of his siblings had died suddenly of presumed cardiac causes in early adulthood (ages 32, 38, and 41 years), none with established diagnoses.

### Initial assessment and critical electrocardiographic documentation

During ongoing symptoms at the primary center, a full 12-lead ECG was obtained (Figure 1). This tracing revealed the following:

• Initiation of tachycardia by a premature atrial complex.
• Narrow-complex tachycardia at approximately 220 beats/min, consistent with AVNRT.
• An interpolated extrasystole during sinus rhythm recovery, demonstrating a narrow QRS morphology identical to baseline conducted beats, without a compensatory pause or visible retrograde P waves. These features were most consistent with a Hisian extrasystole rather than typical junctional ectopy, as the latter would be expected to reset the sinus node or produce retrograde atrial activation.^6^
• Beat-to-beat QRS amplitude variability across successive complexes during tachycardia, suggesting dynamic changes in ventricular activation possibly reflecting subtle alterations in Purkinje-muscle conduction or functional block.^7^
• Atrial activity preceding QRS complexes in lead aVL during tachycardia.

**Figure 1:**
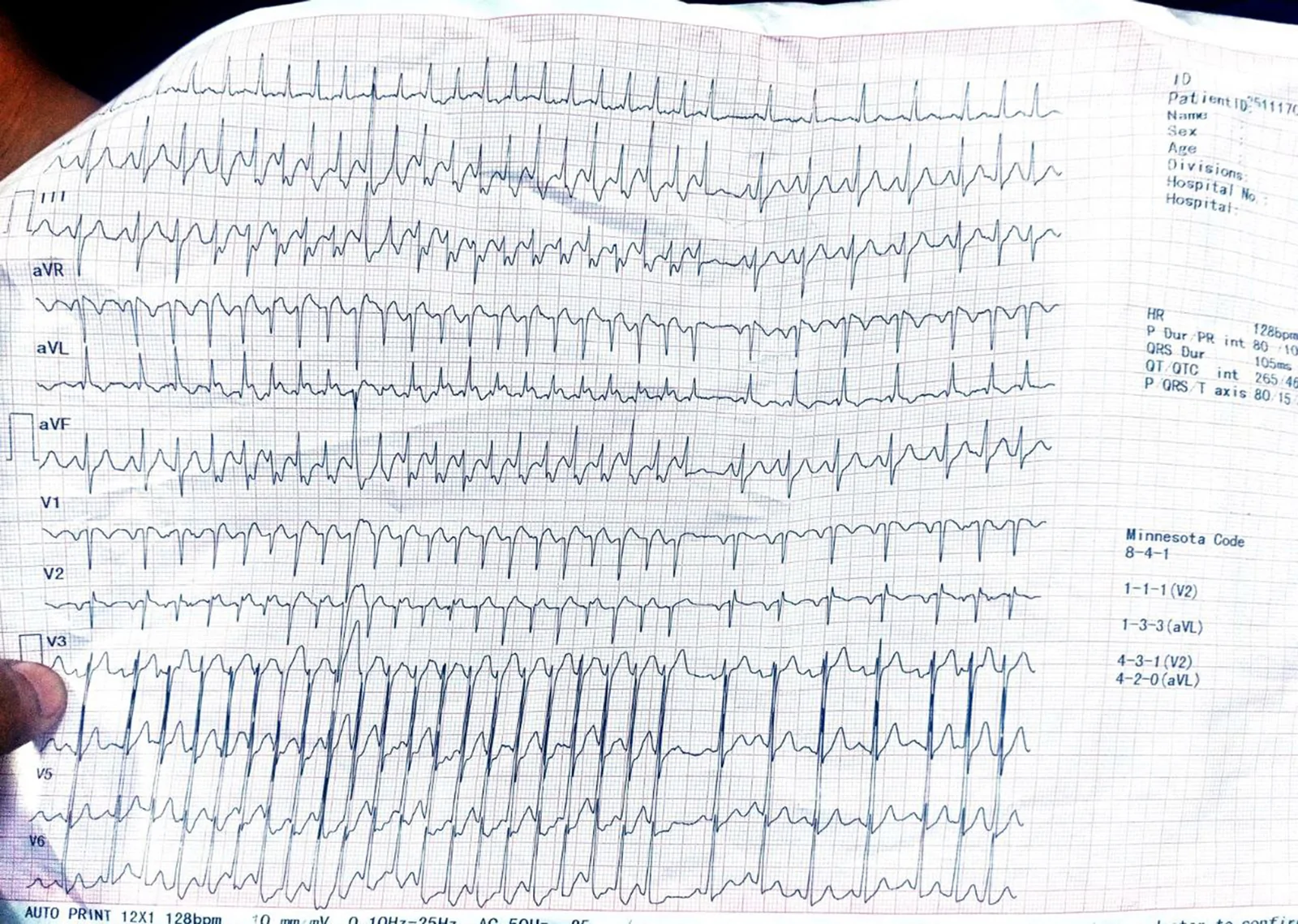
Symptomatic 12-Lead Electrocardiogram Initiation of narrow-complex tachycardia (∼220 beats/min) by a premature atrial complex (PAC). Interpolated Hisian extrasystole (arrowhead) during sinus rhythm recovery. Beat-to-beat QRS amplitude variability during tachycardia (brackets).

The patient was immediately transferred to a tertiary care emergency department. On arrival, the tachyarrhythmia had spontaneously terminated, and no repeat symptomatic ECG was obtainable.

### Physical examination and vital signs

Postconversion examination revealed an alert, oriented man. Vital signs: blood pressure 160/80 mm Hg, heart rate 60 beats/min, respiratory rate 20 breaths/min, and oxygen saturation 98% on room air. Cardiac auscultation revealed regular rhythm without murmurs, rubs, or gallops. Lung fields were clear. No peripheral edema or signs of heart failure were present.

### Investigations

Laboratory Studies: Complete blood count, electrolytes, renal function, and random glucose were within normal limits. Cardiac biomarkers (troponin-I, CK-MB) were negative, excluding acute myocardial infarction.

Chest Radiography: Posteroanterior view demonstrated normal cardiomediastinal silhouette and clear lung fields.

Transthoracic Echocardiography: An initial echocardiogram performed at the referring center was interpreted as showing "basal inferoseptal hypokinesia" and labeled "ischemic." Expert re-review at the tertiary center demonstrated the following:

• No coronary territorial wall motion abnormality
• Mild global left ventricular systolic dysfunction (left ventricular ejection fraction ∼45%)
• Subtle, nonterritorial segmental hypokinesia
• Normal right ventricular size and function
• Trivial tricuspid regurgitation only

The initial ischemic interpretation was deemed an overcall; findings were reclassified as nonspecific, possibly representing early cardiomyopathic changes.

Cardiac Magnetic Resonance (CMR): CMR with late gadolinium enhancement (LGE) was strongly recommended for further substrate characterization given the concerning clinical presentation and family history. However, at the time of manuscript submission, CMR has not yet been performed due to institutional scheduling logistics. This represents an important limitation, as definitive assessment for structural fibrosis, fibrofatty replacement, or other arrhythmogenic substrate awaits CMR confirmation.

### Resting electrocardiogram (postconversion)

• Sinus rhythm with short PR interval (∼100 ms) without delta waves
• Distinct atrial echo waves appearing as small, inverted P waves following the T wave in inferolateral leads (Figure 2)

**Figure 2:**
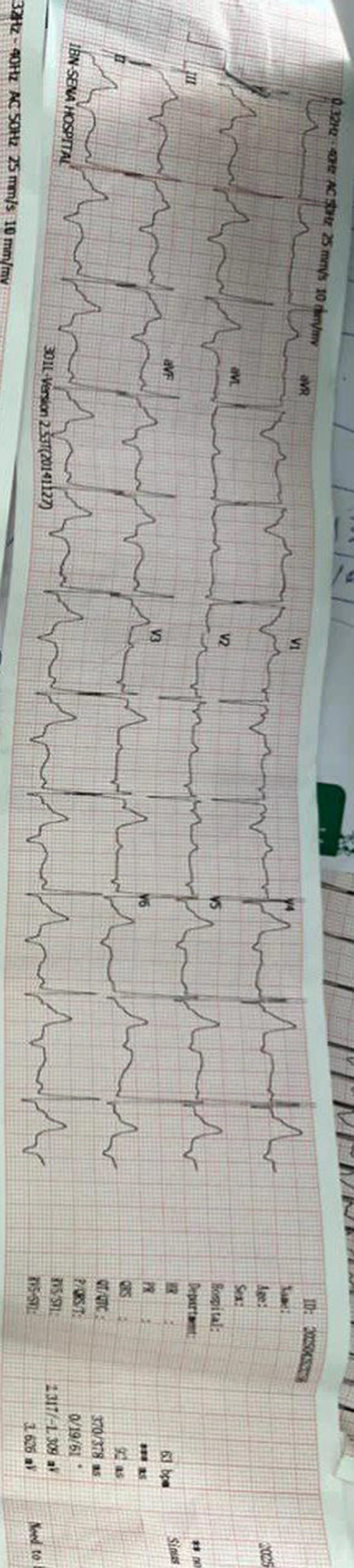
Resting Electrocardiogram Post Conversion Sinus rhythm with short PR interval (∼100 ms) without delta waves. Retrograde atrial echo waves appear as small, inverted deflections following the T-wave in inferolateral leads (II, III, aVF, V_5_-V_6_) and upright deflections in aVR. These findings are consistent with dual AV nodal physiology.

**Visual Summary:**
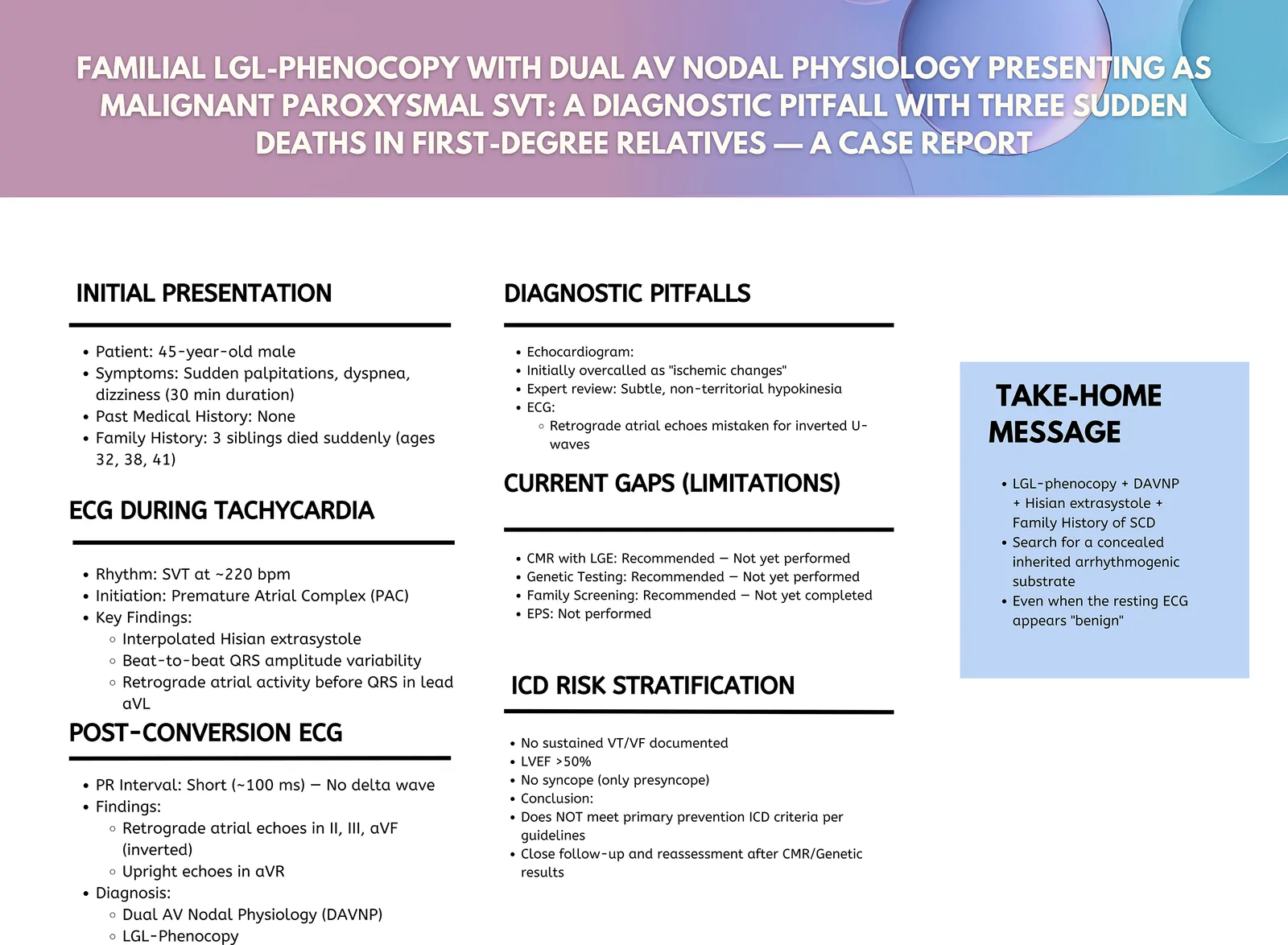
Case Summary Clinical presentation and family history (3 sudden deaths). Electrocardiographic red flags: short PR interval, dual AV nodal physiology, Hisian extrasystole, and rapid SVT at 220 beats/min. Diagnostic considerations and management algorithm including CMR, genetic testing, family screening, and ICD candidacy reassessment. AV = atrioventricular; CMR = cardiac magnetic resonance; ECG = electrocardiogram; EP = electrophysiology; ICD = implantable cardioverter-defibrillator; LGE = late gadolinium enhancement; LGL = Lown-Ganong-Levine; LVEF = left ventricular ejection fraction; SCD = sudden cardiac death; SVT = supraventricular tachycardia; VF = ventricular fibrillation; VT = ventricular tachycardia.
• Upright atrial deflections in lead aVR
• These findings were consistent with an LGL-phenocopy pattern with underlying DAVNP

### Hospital course and working diagnosis

The patient remained hemodynamically stable and required no acute intervention. The working diagnosis was premature atrial complex–triggered AVNRT in the setting of LGL-phenocopy with DAVNP, with electrocardiographic features (Hisian extrasystole, QRS variability) and family history strongly suggestive of an underlying inherited arrhythmogenic cardiomyopathy.

A multidisciplinary team recommended expedited evaluation including CMR with LGE, extended Holter monitoring, comprehensive genetic testing for arrhythmogenic cardiomyopathy genes (DSP, PKP2, DSG2, LMNA, RYR2, etc), and referral for family cascade screening. Given the malignant family history, primary prevention strategies including potential implantable cardioverter-defibrillator (ICD) insertion were discussed.

## Discussion

This case exemplifies a critical diagnostic convergence where a seemingly benign electrocardiographic variant heralded a lethal familial arrhythmogenic condition. Several key teaching points emerge.

1. The Short PR Phenotype: From Benign Variant to Potential Red Flag

The historical LGL syndrome has been rightfully deconstructed, with contemporary practice favoring descriptive terminology.^2^ However, this conceptual shift should not lead to complacency. When a short PR pattern coexists with other electrocardiographic aberrations—particularly His-Purkinje system abnormalities—and a significant family history, it transitions from incidental finding to diagnostic clue.^8^ Our patient's presentation underscores that the "LGL-phenocopy" label should prompt, not preclude, thoughtful evaluation in the proper clinical context.

2. DAVNP: A Substrate for More Than Benign SVT

DAVNP is the established substrate for typical AVNRT, usually considered a benign arrhythmia with excellent treatment outcomes.^3^ However, when DAVNP presents with unusual features—such as extremely rapid ventricular rates (∼220 beats/min), interpolated Hisian extrasystoles, and beat-to-beat QRS variability—it should raise suspicion for an abnormal underlying myocardial or conduction system substrate.^7^ The presence of these features in our patient suggests that the DAVNP was not an isolated electrical anomaly but part of a broader pathological process affecting the cardiac conduction system.

3. Hisian Extrasystoles and QRS Variability: Electrocardiographic Sentinels of Purkinje Disease

The interpolated Hisian extrasystole documented in our patient represents a rare but significant finding. His-Purkinje system ectopy is increasingly recognized as a potential marker for underlying structural heart disease, particularly arrhythmogenic cardiomyopathies with conduction system involvement.^9^ Similarly, the beat-to-beat QRS amplitude variability observed during tachycardia suggests dynamic changes in ventricular activation, possibly due to varying degrees of Purkinje-muscle conduction impairment or functional conduction block.

Electrocardiographic Recognition of Hisian Extrasystole: The diagnosis of a Hisian extrasystole was inferred from surface ECG characteristics rather than intracardiac recordings. The premature beat demonstrated a narrow QRS complex identical to baseline morphology, indicating activation through the native His-Purkinje system without aberrancy. The fixed coupling interval and interpolated nature—occurring between 2 sinus-conducted beats without a compensatory pause—are atypical for junctional ectopy, which typically resets the sinus node or produces a full compensatory pause due to retrograde atrial penetration.^6^ Importantly, the absence of a clearly discernible retrograde P wave further distinguishes this finding from typical junctional premature beats, where retrograde atrial activation is often visible.^7^ The ectopic impulse appeared to interact with ongoing AV nodal conduction, creating a pattern of concealed conduction rather than fully expressed retrograde penetration—a phenomenon well-described with Hisian extrasystoles exhibiting concealed retrograde block.^10^

We acknowledge that intracardiac electrophysiologic study remains the gold standard for definitive localization, and in its absence, junctional ectopy cannot be entirely excluded; however, the constellation of surface ECG features—particularly the interpolated nature, lack of retrograde P waves, and fixed coupling—strongly favors a Hisian origin in this clinical context.

4. The Imperative of Early 12-Lead ECG Documentation

The diagnostic pivot in this case was the 12-lead ECG obtained during symptoms at the primary health center. Without this tracing, the critical findings—Hisian extrasystole, QRS variability, and precise tachycardia mechanism—would have been missed. This underscores a fundamental principle in arrhythmia management: the first medical contact during symptoms represents the golden window for diagnostic electrocardiography.^11^ Systems must prioritize obtaining and preserving such tracings, ensuring they accompany patients during transfer.

5. Familial Sudden Death: The Ultimate Red Flag

Three sudden deaths among first-degree relatives constitute a powerful risk signal that should dominate clinical decision-making. Current guidelines for inherited primary arrhythmia syndromes emphasize that such family history significantly increases pretest probability and warrants aggressive evaluation.^12^ In our patient, this history transformed the interpretation of otherwise subtle findings, justifying comprehensive genetic and phenotypic assessment.

6. Diagnostic Pitfalls: From Imaging Overcall to Electrocardiographic Misinterpretation

This case illustrates 2 common diagnostic errors:

Echocardiographic overinterpretation: The initial "ischemic" reading based on subtle wall motion abnormalities nearly diverted the diagnostic pathway. Expert re-review was essential to correct this misclassification.
Electrocardiographic underrecognition: The retrograde atrial echo waves were initially misidentified as U-wave abnormalities. Recognition of these low-amplitude potentials requires systematic ECG analysis and understanding of DAVNP manifestations.

## Differential Diagnosis: A Balanced Consideration

Although the constellation of findings in our patient points toward an underlying inherited arrhythmogenic cardiomyopathy, a rigorous differential diagnosis must consider alternative explanations. This balanced approach enhances scientific rigor and acknowledges the limitations inherent in a case without definitive genetic or advanced imaging confirmation.

The following diagnostic possibilities warrant consideration:

1. Familial AVNRT without structural disease: DAVNP can be an isolated familial trait, and AVNRT itself may occur in structurally normal hearts.^3^ However, this explanation would not account for the Hisian extrasystoles, QRS variability, or the striking family history of SCD in 3 first-degree relatives. Isolated AVNRT, even when familial, does not typically confer such malignant risk.
2. Laminopathy (LMNA-related disease): LMNA mutations often present with conduction system abnormalities and atrial arrhythmias before developing dilated cardiomyopathy and ventricular arrhythmias.^13^ Although our patient's short PR interval differs from the progressive conduction delay typically seen in laminopathy, the family history of SCD and evidence of conduction system involvement remain compatible. Laminopathy can manifest with variable phenotypes, and early stages may present with isolated electrical abnormalities.
3. DSP cardiomyopathy: DSP mutations cause a distinct form of arrhythmogenic cardiomyopathy with left ventricular involvement, conduction abnormalities, and high SCD risk, sometimes preceding overt structural changes.^5^ This diagnosis is highly consistent with our patient's presentation: left-dominant phenotype, conduction system involvement (Hisian extrasystole, DAVNP), and profound family history of SCD. Pending CMR and genetic testing will be critical to confirm or refute this hypothesis.
4. Other arrhythmogenic cardiomyopathy variants: Mutations in genes such as PKP2, DSG2, DSC2, and PLN can produce overlapping phenotypes with variable left ventricular involvement and conduction disease.^4^ Without genetic data, these remain in the differential.
5. Ion channel disorders: SCN5A mutations can produce a spectrum of phenotypes including conduction delay, short PR intervals, and increased arrhythmic susceptibility.^14^ Brugada syndrome and early repolarization syndrome were considered but deemed less likely given the absence of characteristic ECG patterns (coved-type ST elevation in right precordial leads, early repolarization pattern in inferolateral leads). However, concealed or nondiagnostic presentations cannot be entirely excluded.
6. Cardiac sarcoidosis: Cardiac sarcoidosis can manifest with conduction abnormalities, atrial and ventricular arrhythmias, and LGE on CMR.^15^ This diagnosis is less consistent with the family history of SCD in multiple siblings, although coincidental familial clustering of sarcoidosis is theoretically possible.
7. Cryptographic inherited arrhythmia syndromes: Some families with malignant SCD history remain genetically elusive despite comprehensive testing. Negative genetic testing would not exclude an inherited substrate, as current panels do not capture all pathogenic variants.

In synthesizing this differential, we emphasize that in the absence of definitive structural (CMR) and genetic confirmation, the current interpretation should be viewed as a working hypothesis rather than a final etiologic diagnosis. The weight of evidence—particularly the electrocardiographic red flags combined with the profound family history—strongly suggests an inherited arrhythmogenic substrate, with DSP cardiomyopathy and laminopathy being leading contenders pending further investigation.

## Management Implications and Future Directions

### This case mandates a structured management approach

#### Advanced phenotyping

CMR with LGE and T1 mapping is essential to detect fibrofatty replacement, myocardial edema, or subtle fibrosis characteristic of arrhythmogenic cardiomyopathies.^4^ Although pending, completion of CMR remains a priority.

#### Genetic testing

Comprehensive testing for inherited arrhythmia and cardiomyopathy genes has been strongly recommended given the striking family history. Although not performed to date, identification of a pathogenic variant would confirm the diagnosis, guide family cascade screening, and inform risk stratification.

#### Family cascade screening

Systematic cardiology evaluation of all first-degree relatives has been advised and should include 12-lead ECG, echocardiography, Holter monitoring, and consideration of provocative testing where appropriate. These evaluations are currently pending and represent essential next steps.

#### Risk stratification and ICD consideration

The question of ICD candidacy requires careful consideration of current guideline recommendations. According to the 2017 American Heart Association/American College of Cardiology/Heart Rhythm Society Guideline^11^ and the 2022 European Society of Cardiology Guidelines,^12^ primary prevention ICD insertion is recommended in specific high-risk populations even in the absence of documented sustained ventricular arrhythmias, provided certain criteria are met.

Our patient does not currently fulfill definitive Class I indications for primary prevention ICD. Specifically: 1) no sustained ventricular arrhythmias have been documented; 2) left ventricular ejection fraction is preserved (>50%); and 3) no syncope of suspected arrhythmic etiology has occurred (the patient experienced presyncope/dizziness during tachycardia, not true syncope).

However, several high-risk features warrant careful consideration:

• A profound family history of SCD in 3 first-degree relatives, recognized as a major risk modifier in arrhythmogenic cardiomyopathy.^4^ Electrocardiographic evidence of conduction system disease (Hisian extrasystole, short PR phenotype, DAVNP with extremely rapid SVT at ∼220 beats/min)
• Potential early structural disease pending CMR confirmation

The combination of these features places this patient in a "gray zone" where individualized risk stratification is essential. Current guidelines acknowledge that in patients with arrhythmogenic cardiomyopathy and multiple risk factors, ICD insertion may be considered (Class IIb) even without documented sustained ventricular arrhythmias.^12^

Therefore, we have revised our position to state: The patient does not currently meet definitive guideline-based criteria for primary prevention ICD insertion. However, the constellation of high-risk features—particularly the malignant family history and electrocardiographic evidence of conduction system disease—warrants close follow-up, further risk stratification (including electrophysiological study and CMR), and reassessment of ICD candidacy pending these results. A shared decision-making approach with the patient, incorporating his values and preferences, is essential.

## Outcome and Follow-Up

The patient was discharged in stable condition with close outpatient follow-up scheduled. He remains asymptomatic at 2-month follow-up, with no recurrent palpitations. He has been referred for elective electrophysiological study and genetic counseling. Family cascade screening has been initiated for first-degree relatives. The patient continues to be monitored pending CMR and genetic testing results.

### Study limitations

This case report has several limitations that should be considered when interpreting the findings:

Cardiac MRI not performed: Although strongly recommended, CMR with LGE has not been obtained. Consequently, definitive assessment for structural fibrosis, fibrofatty replacement, or other arrhythmogenic substrate—which would strongly support an arrhythmogenic cardiomyopathy—is not available. The hypothesis of underlying structural disease remains unconfirmed and awaits advanced imaging.

Genetic testing not performed: Comprehensive genetic testing for inherited arrhythmia and cardiomyopathy genes has not been completed. This precludes definitive etiologic classification, limits our ability to identify a specific molecular substrate, and prevents targeted cascade screening of at-risk family members.

Family screening incomplete: Systematic cardiology evaluation of first-degree relatives has not been undertaken. Therefore, our understanding of familial penetrance, phenotypic expression, and risk distribution within this family remains incomplete.

Absence of intracardiac electrophysiologic study: The diagnosis of Hisian extrasystole was inferred from surface ECG characteristics rather than confirmed with intracardiac recordings. Although the electrocardiographic features strongly favor this interpretation, invasive electrophysiology remains the gold standard for definitive localization.

Single-center experience and limited follow-up: This report represents a single-center experience with limited follow-up data. Long-term outcomes and the natural history of this patient's condition remain unknown.

These limitations reflect real-world challenges in completing comprehensive evaluations, particularly in resource-constrained settings. They should not diminish the clinical significance of the electrocardiographic red flags and malignant family history that strongly suggest an underlying inherited arrhythmogenic substrate. Rather, they highlight the need for continued investigation and should guide future directions for this patient and family. The findings presented herein should be viewed as a working hypothesis guiding further evaluation rather than a final etiologic diagnosis.

## Conclusions

An apparently benign short PR ECG pattern with DAVNP can conceal a life-threatening inherited arrhythmogenic substrate. Recognition of electrocardiographic red flags—particularly Hisian extrasystoles and beat-to-beat QRS variability—in the context of a malignant family history should trigger comprehensive evaluation for underlying cardiomyopathy. This case emphasizes the critical importance of early 12-lead ECG documentation during symptoms and underscores the need for a high index of suspicion when common electrophysiological phenomena present with uncommon features. Pending advanced imaging and genetic results, the findings herein serve as a compelling reminder that the surface ECG often holds keys to deeper, unsuspected pathology.

### Data Availability

This article is a case report and does not include any datasets that can be shared. All clinical details are presented within the manuscript in an anonymized format.

**Take-Home Messages**

• An apparently benign short PR phenotype can mask a lethal heritable substrate. Recognition of electrocardiographic red flags and meticulous family history are paramount.
• Pending CMR and genetic results, this case highlights critical diagnostic pitfalls and the imperative of familial evaluation.

## Funding Support and Author Disclosures

This case report was conducted without any financial support. The authors have reported that they have no relationships relevant to the contents of this paper to disclose.
